# The potential therapeutic effect of quercetin on mitochondrial dysfunction in hepatorenal toxicity induced by aluminum chloride in an experimental rat model

**DOI:** 10.1038/s41598-026-62378-6

**Published:** 2026-08-03

**Authors:** Tasneem N. Hafez, Magda A. Megahed, Bothaina F. Mahmoud, Mohammed Salama, Nesma A. Ghazal

**Affiliations:** 1 Department of Biochemistry, Medical Research Institute, Alexandria University, 165 El-Horreya Avenue, EL-Hadara, POB: 21561, Alexandria, Egypt; 2 Department of Histochemistry and Cell Biology, Medical Research Institute, Alexandria University, Alexandria, Egypt

**Keywords:** Hepatorenal toxicity, Aluminum chloride, Quercetin, Mitochondrial biogenesis, mtDNA-CN, Biochemistry, Diseases, Drug discovery, Medical research, Molecular biology, Physiology

## Abstract

Aluminum is a xenobiotic element known to induce hepatorenal toxicity through mechanisms involving mitochondrial dysfunction, oxidative stress, and inflammation. Quercetin, a dietary flavonoid with potent antioxidant and anti-inflammatory properties, has shown promise as a therapeutic agent. This study aimed to evaluate the potential therapeutic effects of quercetin against aluminum chloride (AlCl₃)-induced hepatorenal toxicity and mitochondrial dysfunction in rats. Hepatorenal toxicity was induced by oral administration of hydrated aluminum chloride (75 mg/kg body weight) daily for six weeks. Quercetin was administered intraperitoneally at a dose of 30 mg/kg body weight daily for four weeks. Biochemical assays, mitochondrial gene expression analysis, and histopathological examinations were conducted to assess the therapeutic effects. Quercetin significantly ameliorated lipid, protein, and DNA oxidation parameters (MDA, AOPPs and 8-OHdG respectively), reduced inflammation marker (TNF-α), and restored mitochondrial biogenesis markers, including PGC-1α, mtTFA and mitochondrial DNA copy number (mtDNA-CN). In addition, Quercetin significantly decreased TNF-α and increased PGC-1α contents at protein levels. Histopathological findings corroborated these results, demonstrating that quercetin improved liver and kidney architecture. These findings suggest that quercetin may serve as a potential therapeutic agent for aluminum-induced hepatorenal toxicity.

## Introduction

Aluminum chloride (AlCl₃) exposure poses a significant environmental and occupational health risk due to its toxic effects on vital organs, particularly the liver and kidneys. The mechanisms underlying AlCl₃-induced toxicity include oxidative stress, mitochondrial dysfunction, and impaired antioxidant defenses^1^**.**

In the liver, AlCl₃ exposure disrupts mitochondrial energy metabolism, elevates serum liver enzymes, and induces histopathological changes such as necrosis and fibrosis**.** Similarly, in the kidneys, AlCl₃ causes tubular necrosis, fibrosis, and epithelial hyperplasia, further exacerbating organ dysfunction^2^**.**

The master regulator of mitochondrial biogenesis, peroxisome proliferator activator receptor gamma-coactivator 1α (PGC-1α), controls the expression of mtTFA which facilitate the transcription, replication of mitochondrial DNA (mtDNA) and mitochondrial biogenesis^3^**.**

Mitochondrial dysfunction is central to the pathogenesis of hepatorenal toxicity, as mitochondria are critical for energy production and cellular homeostasi^4^**.** Disruption of mitochondrial biogenesis contributes to decreased ATP production and increased reactive oxygen species (ROS), leading to cellular damage^5^**. **Following oxidative stress exposure, Nrf2 is phosphorylated and Keap1 becomes inactive. When phosphorylateyod Nrf2 (p-Nrf2) accumulates in the nucleus, it binds with the antioxidant-response element (ARE) and activates a variety of genes, including those that produce transport molecules, detoxifying enzymes, and antioxidants^6^. Extensive evidence highlights a reciprocal regulatory feedback loop between PGC-1α and Nrf2, wherein Nrf2 directly drives mitochondrial biogenesis and cross-talks with PGC-1α to promote lifespan extension^7^.

Quercetin, a naturally occurring flavonoid, has garnered attention for its antioxidant and anti-inflammatory properties. It neutralizes free radicals, reduces oxidative stress, and modulates biological pathways involved in inflammation and mitochondrial function. Recent studies have highlighted its protective effects against renal inflammation, ferroptosis, and apoptosis^8^**.**

Given the pivotal role of mitochondrial dysfunction in AlCl₃ induced toxicity, this study investigates the therapeutic potential of quercetin in mitigating mitochondrial damage and hepatorenal toxicity in an experimental rat model.

## Materials and methods

### Experimental animals

A total number of 32 two months Wistar male albino rats, (100-150g) were used. The animals were obtained from the animal house of Medical Research Institute, Alexandria University, Egypt. Rats were housed in standard cages in well-ventilated rooms (25 ± 2 °C), with a relative humidity of (43 ± 3), with free access to water and food and 12 hours’ light/dark cycle before experimentation.

### Ethical statement

All experiments pursued the standards of the National Institute of Health Guide for the Care and Use of Laboratory Animals (NIH Publications No. 8023, revised 1978) and were performed after the approval of the Institutional Animal Care and Use Committee (IACUC)-Alexandria University, Egypt (Approval No.: AU01223101512). The study also followed ARRIVE guidelines and complied with the National Research Council’s Guide for the Care and Use of Laboratory Animals.

### Induction of hepatorenal toxicity

Hepatorenal toxicity was induced in rats using hydrated aluminum chloride (AlCl_3_.6H_2_O) solution that was given orally at a dose of 75 mg/kg body weight daily for 6 consecutive weeks^9^

### Treatment with quercetin

Quercetin obtained from Sigma Aldrich was administrated intraperitoneally to rats as a powder dissolved in water at a dose of 30 mg/kg body weight daily for 4 weeks^10^**.**

### Experimental designas

The animals were given standard food and water *ad-libitum*. Rats were classified into four groups each group contains 8 rats: **Group I (Control group)**, normal healthy male rats. **Group II (Quercetin control group)**, rats were received a daily intraperitoneal injection of quercetin (30 mg/kg body weight, dissolved in 0.25% v/v DMSO) for 4 weeks^10^**. Group III (Untreated AlCl**_**3**_** group)**, rats were administered aluminum chloride (AlCl_3_.6H_2_O) solution (75 mg/kg body weight /day)^9^ orally for 6 consecutive weeks followed by daily intraperitoneal injections of the DMSO vehicle alone for 4 weeks. **Group IV (Quercetin-treated AlCl**_**3**_** group)**, rats were administered AlCl_3_.6H_2_O solution (75 mg/kg body weight/day) orally for 6 consecutive weeks then treated interperitoneally wih quercetin (30 mg/kg body weight, dissolved in 0.25% v/v DMSO) daily for 4 weeks.

### Collection of samples

After 24 hours from the last administration, rats in all groups were sacrificed under deep anesthesia via isofluoran inhalation. The blood samples were collected from dorsal vein into serum gel separator tubes from each rat. The samples were left f or 20 min at 4^◦^C, centrifuged at 3000 xg for 10 minutes using Hettich Zentrifugen Tuttlingen centrifuge to obtain serum. Sera were stored at −80^◦^C until used for assessment liver function tests (ALT, AST, ALP, total bilirubin), kidney function tests (urea, creatinine), lipid profile parameters (total cholesterol, triglycerides, HDL-C, LDL-C), and advanced oxidation protein products (AOPPs).

The excised liver and kidney tissues were rinsed with saline and then divided into two halves**. The first half** was divided into two parts. **First part** of excised tissue was homogenized in phosphate buffer saline (PBS) pH 7.4 in the ratio of 1:10 (0.125 gm of tissue in 1.25 ml PBS). The homogenate was used for the determination of malondialdehyde (MDA) while the second aliquot was centrifuged at 10000 rpm, at 4°C for 20 minutes and the obtained supernatants were used for the determination of 8-hydroxy guanidine (8-OHdG), Peroxisome proliferator activator receptor gamma-coactivator 1α (PGC-1 α) and tumor necrosis factor alpha (TNFα) by ELISA. **Second part** of excised tissue was used for the extraction of total RNA for Quantitative Real Time-Polymerase Chain Reaction (qRT-PCR) analysis for assessment of gene expression of PGC-1 α, mitochondrial transcription factor A (mtTFA), nuclear factor-erythroid 2-related factor 2 (Nrf2) and TNF-α and extraction of DNA for determination of mtDNA-CN. **Second half** of liver and kidney tissues was fixed in 10 % buffered formalin for histological examination.

### Histopathological examination

Following necropsy, liver and kidney specimens were immediately fixed in phosphate-buffered formalin (10%, pH 7.4) for at least 24 hours which were then processed using conventional paraffin embedding technique^11^ Sections of 5 μm thick were sliced, mounted on slides deparaffinated in xylene and rehydrated using decreasing concentrations of ethanol. Slides were stained with hematoxylin and eosin (H&E) for routine histopathological setting. Stained sections were blindly evaluated using light microscope (Leica, DM500) and photographed at a magnification of ×400 using a digital camera (EC3, Leica, Germany). The **histopathological staging (or scoring)** was done as a **semi-quantitative assessment**. This scoring is based on a standard **0 to 3 scale** , evaluating the **percentage of tissue damage or alteration** observed across multiple microscopic fields (usually graded as: 0 = Normal, 1 = Mild [<25%], 2 = Moderate [25–50%], 3 = Severe [>50%])**. For Liver Tissue Staging Parameters,** the scoring for liver slides evaluates the degree of tissue injury based on two main criteria: Hepatocyte vacuolation and degeneration**. For Kidney Tissue Staging Parameters,** the renal scoring evaluates the deformity, shrinkage, or congestion of the glomeruli and the widening/loss of Bowman’s space.

### Serum parameters measurements

The blood samples were obtained and assayed for liver function parameters according to the manufacturer’s instructions using serum ALT Bio-Med Diagnostic INC (USA) kit (**ALT Cat. No.: 1200**)^12^**,** serum AST Bio-Med Diagnostic INC (USA) kit (**AST Cat. No.: 1202)**^12^, serum ALP Bio-Med Diagnostic INC (USA) kit (**ALP Cat. No.: 101090)**^13^**,** and serum total bilirubin spectrum Diagnostic (Germany) kit (**Cat. No.: 222 001)**^14^**.**

Also, kidney function parameters were assayed according to the manufacturer’s instructions using serum urea Bio-Med Diagnostic INC (USA) kit (**Cat. No.: IFUFCC40**)^15^**,** and serum creatinine Bio-Med Diagnostic INC (USA) kit (**Cat. No.: IFUFCC09)**^16^**.**

In addition, lipid profile was investigated by using cholesterol Agappe Diagnostic LTD (India) kit^17^**,** serum triglycerides Agappe Diagnostic LTD (India) kit^13^ and serum HDL-C level Agappe Diagnostic LTD (India) kit^18^**.** Serum LDL–C was calculated^19^**.**

Determination of AOPPs levels according to the manufacturer’s instructions using AOPPs ELISA kit (**Cat. No.: CSBEQ027429RA**)^20^**.**

### Determination of malondialdehyde (MDA) content

Malondialdehyde was determined **according to the method of**^21^**.** The tissue samples were heated with thiobarbituric acid (TBA) at low pH. The resulting pink chromogen has a maximal absorbance at 532 nm**.**

### Protein content determination of 8-OHdG, PGC1α, and TNF-α by ELISA.

The content of rat 8-OHdG, PGC1α, and TNF-α in the samples were measured by ELISA kit (**Cat. No.: CSB-E10526r**)^22^, (**Cat. No.: MBS1600735**)^23^**,** and (**Cat. No.: CSB-E11987r**)^24^**.** respectively.

### Determination of total protein contents

A modification method of **Lowry *et al.***^25^ was used for the determination of protein in the samples.

### Gene expression analysis

Thirty mg of kidney tissues were used for total RNA extraction using Gene Direx Kit (USA) (Cat. No.: NA021-0100) according to the manufacturer’s instructions. The concentration and integrity of extracted RNA were checked using nanodrop. The reverse transcription of the extracted RNA was performed by Viva cDNA Synthesis Kit (vivantis) according to the manufacturer instructions. The tissues expression of **PGC-1α, mtTFA, Nrf2, and TNF-α** were quantified in the cDNA by CFX Maestro™ Software (Bio-Rad, USA) using Rotor-Gene SYBR Green PCR Kit **(Qiagen®, Germany).** The housekeeping gene **18S rRNA** was used as a reference gene for normalization. The primers used for the determination of rat genes are presented in Table 1**.** The relative change in mRNA expression in samples was calculated using the 2^-ΔΔCt^ method^26^**.**

**Table 1:** Primer sets of PGC-1α, mtTFA, Nrf2, TNF-α, and 18S rRNA.

**Gene**	**Accession number**	**primer sequence**	
**18S rRNA** **(**reference gene)	**NR_046237.2**	**F:**	5'-GTAACCCGTTGAACCCCATT-3'
		**R:**	5'-CAAGCTTATGACCCGCACTT-3'
**PGC-1α**	**NM_031347.1**	**F:**	5'- GTGCAGCCAAGACTCTGTATGG -3'
		**R:**	5'- GTCCAGGTCATTCACATCAAGTTC -3'
**mtTFA**	**NM_031326.2**	**F:**	5'-CCCACAGAGAACAGAAACAG-3'
		**R:**	5'-CCCTGGAAGCTTTCAGATACG-3'
**Nrf2**	**NM_017008.4**	**F:**	5'-CGAGATATACGCAGCAGGAGAGGTAAG-3'
		**R:**	5'-GCTCGACAATGTTCTCCAGCTT-3'
**TNF-α**	**NM_012675.3**	**F:**	5-TGGGCTCCCTCTCATCAGTTC- 3
		**R:**	5-TCCGCTTGGTGGTTTGCTAC- 3

### Mitochondrial DNA copy number determination

A qRT-PCR assay was developed to estimate relative mtDNA copy number (mtDNA-CN) by comparing PCR amplicons of mitochondrial DNA to a single nuclear gene. Following genomic DNA isolation, specific primer pairs for mtDNA and nuclear PGC-1α were used in equal PCR cycles to calculate the mtDNA signal relative to nuclear DNA. The mtDNA content is expressed as the ratio Ct (mtDNA)/Ct (nDNA), where lower Ct values indicate higher template concentration. This ratio demonstrates that increasing Ct values correlate with a decrease in mtDNA per cell, ultimately representing mtDNA-CN as log R, where R=2 –ΔCt and ΔCt= Ct mtDNA – Ct nDNA^27^ Table 2.

**Table 2:** Primers for nuclear PGC-1α and mtDNA for qRT-PCR.

**Gene Name**	**Accession number**	**Primer sequence**	
**Nuclear PGC-1α**	**NM_031347.1**	**F**	5'-ATGAATGCAGCGGTCTTAGC-3'
		**R**	5'-AACAATGGCAGGGTTTGTTC-3'
**mtDNA**	**X14848.1**	**F**	5'-ACACCAAAAGGACGAACCTG-3'
		**R**	5'- ATGGGGAAGAAGCCCTAGAA-3'

### Statistical analysis

Data were analyzed using SPSS software package version 18.0 (SPSS Chicago, IL, USA). The data were expressed as means ± SD and analyzed using a one- way analysis of variance (ANOVA) and followed by post hoc Tukey test to compare the mean values between and within treated groups compared to untreated and control groups. Differences were considered statistically significant at *p* value < 0.05. Correlation studies were performed using Pearson’s correlation coefficient^28^.

## Results

### Liver function tests

The AlCl_3_ exposure significantly increased serum ALT, AST, ALP activities and total bilirubin levels compared to controls (p ≤ 0.05). Treatment with quercetin significantly reduced these parameters, though not to control levels (p ≤ 0.05). The quercetin control group exhibited lower ALP and total bilirubin levels compared to the untreated AlCl_3_ group, indicating quercetin’s protective role in liver function (Table 3**)**.

**Table 3:** Statistical analysis of serum liver function tests in the different studied groups.

**Groups**	**Control**	**Quercetin control**	**Untreated AlCl**_**3**_** group**	**Quercetin-treated AlCl**_**3**_** group**
**Serum ALT (U/L)**	34.0 ± 2.51	36.37 ± 1.06	65.50^ab^ ± 2.51	49.25^abc^ ± 1.28
**Serum AST (U/L)**	122.5 ± 1.77	124.5 ± 1.93	188.0^ab^ ± 3.34	160.0^abc^ ± 2.73
**Serum ALP (U/L)**	82.0 ± 4.0	76.50^a^ ± 2.07	99.75^ab^ ± 3.24	74.0^ac^ ± 2.67
**Total bilirubin** **(mg/dl)**	0.41 ± 0.02	0.37^a^ ± 0.03	0.62^ab^ ± 0.02	0.44^abc^ ± 0.02

n= **8 replicas** in each groupData was expressed using Mean ± SD.

**By using One way ANOVA test**, pairwise comparison between each 2 groups were done using **Post Hoc Test (Tukey)**

p: p value for comparing between the studied groups

*: Statistically significant at p ≤ 0.05

a: Significant with **Control**

b: Significant with **Quercetin control**

c: Significant with **AlCl3 (untreated).**

### Kidney function tests

As shown in (Table 4**)**, AlCl_3_ exposure significantly raised urea and creatinine levels (p ≤ 0.05). Quercetin treatment reduced these levels significantly compared to untreated AlCl3-exposed rats, though levels remained elevated compared to the control group (p ≤ 0.05). Quercetin alone showed no adverse effects on kidney function.

**Table 4:** Statistical analysis of serum kidney function tests in the different studied groups.

**Groups**	**Control**	**Quercetin control**	**Untreated AlCl**_**3**_	**Quercetin-treated AlCl**_**3**_** group**
**Urea (mg/dl)**	26.0 ± 1.07	28.0 ± 1.60	44.25^ab^ ± 2.25	31.50^abc^ ± 0.93
**Creatinine (mg/dl)**	0.39 ± 0.02	0.34^a^ ± 0.01	0.69^ab^ ± 0.03	0.52^abc^ ± 0.04

n= **8 replicas** in each groupData was expressed using Mean ± SD.

**By using One way ANOVA test**, pairwise comparison bet. each 2 groups were done using **Post Hoc Test (Tukey)**

p: p value for comparing between the studied groups

^*^: Statistically significant at p ≤ 0.05

a: Significant with **Control**

b: Significant with **Quercetin control**

c: Significant with **AlCl3 (untreated).**

### Lipid profile parameters

As seen in (Table 5**),** Total cholesterol, triglycerides, and LDL-C levels were significantly elevated in AlCl3-exposed rats, while HDL-C levels were significantly decreased (p ≤ 0.05). Quercetin treatment significantly reduced cholesterol, triglycerides, and LDL-C levels while improving HDL-C levels, restoring parameters closer to control values (p ≤ 0.05).

**Table 5:** Statistical analysis of lipid profile parameters in the different studied groups.

**Groups**	**Control**	**Quercetin control**	**Untreated AlCl**_**3**_	**Quercetin-treated AlCl**_**3**_** group**
**Total cholesterol (mg/dl)**	71.50 ± 1.31	73.0 ± 1.07	97.25^ab^ ± 3.15	69.0^bc^ ± 2.07
**Triglycerides (mg/dl)**	87.0 ± 2.73	87.0 ± 5.81	108.6^ab^ ± 3.85	95.0^c^ ± 9.34
**HDL- cholesterol (mg/dl)**	42.37 ± 1.06	43.0 ± 1.07	26.50^ab^ ± 1.51	38.75^abc^ ± 2.38
**LDL- cholesterol (mg/dl)**	11.85 ± 0.89	12.60 ± 1.15	50.0^ab^ ± 3.99	15.25^c^ ± 2.86

n= **8 replicas** in each groupData was expressed using Mean ± SD.

**By using One way ANOVA test**, pairwise comparison bet. each 2 groups were done using **Post Hoc Test (Tukey)**

p: p value for comparing between the studied groups

^*^: Statistically significant at p ≤ 0.05

a: Significant with **Control**

b: Significant with **Quercetin control**

c: Significant with **AlCl3 (untreated).**

### Serum advanced oxidation protein products (AOPPs)

As seen in **(**Table 6**),** The AlCl_3_ exposed group exhibited a significantly higher AOPPs level as compared to control groups (p ≤ 0.05). Quercetin treatment significantly reduced AOPPs though it remained higher than the control group (p ≤ 0.05).

**Table 6:** Statistical analysis of serum AOPPs levels (nmol/ml) in the different studied groups.

**Groups**	**Control**	**Quercetin control**	**Untreated AlCl**_**3**_	**Quercetin-treated AlCl**_**3**_** group**
**AOPPs (nmol/ml)**	110.0 ± 6.19	103.5^a^ ± 4.81	224.8^ab^ ± 4.40	142.8^abc^ ± 2.82

n= **8 replicas** in each groupData was expressed using Mean ± SD.

**By using One way ANOVA test**, pairwise comparison bet. each 2 groups were done using **Post Hoc Test (Tukey)**

p: p value for comparing between the studied groups

^*^: Statistically significant at p ≤ 0.05

a: Significant with **Control**

b: Significant with **Quercetin control**

c: Significant with **AlCl3 (untreated).**

### Hepatic and renal Malondialdehyde (MDA)

As seen in (Table 7**),** The AlCl_3_ exposed group exhibited a significantly higher MDA level as compared to control groups (p ≤ 0.05). Quercetin treatment significantly reduced MDA though it remained higher than the control group (p ≤ 0.05).

**Table 7:** Statistical analysis of MDA contents (nmol/mg protein) in the different studied groups.

**Groups**		**Control**	**Quercetin control**	**Untreated AlCl**_**3**_	**Quercetin-treated AlCl**_**3**_** group**
**MDA (nmol/mg protein)**	**Liver**	21.25 ± 3.15	14.25^a^ ± 2.55	59.0^ab^ ± 3.59	34.0^abc^ ± 4.21
	**Kidney**	2.65 ± 0.40	2.33 ± 0.28	4.28^ab^ ± 0.40	3.18^abc^ ± 0.25

n= **8 replicas** in each groupData was expressed using Mean ± SD.

**By using One way ANOVA test**, pairwise comparison bet. each 2 groups were done using **Post Hoc Test (Tukey)**

p: p value for comparing between the studied groups

^*^: Statistically significant at p ≤ 0.05

a: Significant with **Control**

b: Significant with **Quercetin control**

c: Significant with **AlCl3 (untreated).**

### Hepatic and renal 8-hydroxy guanosine (8-OHdG) in rats

As seen in (Table 8), The AlCl_3_ exposed group exhibited a significantly higher 8-OHdG level as compared to control groups (p ≤ 0.05). Quercetin treatment significantly reduced 8-OHdG though it remained higher than the control group (p ≤ 0.05).

**Table 8:** Statistical analysis of hepatic and renal 8-OHdG contents (ng/mg protein) in the different studied groups.

Groups		Control	Quercetin control	Untreated AlCl3	Quercetin-treated AlCl3 group
8-OHdG (ng/mg protein)	Liver	90.0 ± 4.66	82.75^a^ ± 3.65	160.5^ab^ ± 3.74	127.5^abc^ ± 3.16
	Kidney	119.3 ± 4.71	118.0 ± 3.21	180.3^ab^ ± 4.62	132.3^abc^ ± 3.0

n= **8 replicas** in each groupData was expressed using Mean ± SD.

**By using One way ANOVA test**, pairwise comparison bet. each 2 groups were done using **Post Hoc Test (Tukey)**

p: p value for comparing between the studied groups

^*^: Statistically significant at p ≤ 0.05

a: Significant with **Control**

b: Significant with **Quercetin control**

c: Significant with **AlCl3 (untreated).**

### Hepatic and renal peroxisome proliferator activator receptor gamma-coactivator 1α (PGC-1α) contents in rats

As shown in (Table 9), the AlCl_3_ exposed group exhibited a significantly hepatic and renal lower PGC-1α contents as compared to control and quercetin control groups (p ≤ 0.05). Quercetin control rats showed a statistically significant hepatic reduction but renal elevation in PGC-1α contents as compared to control rats (p ≤ 0.05). AlCl_3_ exposed group treated with quercetin showed a statistically significant hepatic elevation in PGC-1α contents as compared to untreated AlCl_3_ exposed rats but statistically significant decline in hepatic PGC-1α as compared to control and quercetin control groups (p ≤ 0.05). In case of renal tissue, there was statistically significant elevation in PGC-1α contents in quercetin treated AlCl_3_ group as compared to untreated AlCl_3_ group (p ≤ 0.05), but not significant difference compared to control and quercetin control groups.

**Table 9:** Statistical analysis of hepatic and renal PGC-1α content (ng/mg protein) in the different studied groups.

Groups		Control	Quercetin control	Untreated AlCl3	Quercetin-treated AlCl3 group
PGC-1α (ng/mg protein)	Liver	8.70 ± 0.72	7.23^a^ ± 0.30	3.80^ab^ ± 0.36	5.78^abc^ ± 0.25
	Kidney	10.57 ± 0.89	12.03^a^ ± 1.07	5.72^ab^ ± 0.64	11.13^c^ ± 0.18

n= **8 replicas** in each groupData was expressed using Mean ± SD.

**By using One way ANOVA test**, pairwise comparison bet. each 2 groups were done using **Post Hoc Test (Tukey)**

p: p value for comparing between the studied groups

^*^: Statistically significant at p ≤ 0.05

a: Significant with **Control**

b: Significant with **Quercetin control**

c: Significant with **AlCl3 (untreated).**

### Hepatic and renal tumor necrosis factor alpha (TNF-α) contents in rats

As shown in (Table 10), the AlCl_3_ exposed group exhibited a significantly hepatic and renal higher TNF-α contents as compared to control and quercetin control groups (p ≤ 0.05). AlCl_3_ exposed group treated with quercetin showed a statistically significant hepatic and renal reduction in TNF-α contents as compared to untreated AlCl_3_ exposed rats but statistically significant increased compared with control and quercetin control group (p ≤ 0.05).

**Table 10:** Statistical analysis of hepatic and renal TNF-α contents (pg/mg protein) in the different studied groups.

**Groups**		**Control**	**Quercetin control**	**Untreated AlCl**_**3**_	**Quercetin-treated AlCl**_**3**_** group**
**TNF-α (pg/mg protein)**	**Liver**	22.75 ± 2.12	18.75 ± 3.33	68.0^ab^ ± 4.0	35.75^abc^ ± 2.87
	**Kidney**	13.25 ± 1.49	15.0 ± 1.60	29.0^ab^ ± 2.73	17.88^abc^ ± 1.81

n= **8 replicas** in each groupData was expressed using Mean ± SD.

**By using One way ANOVA test**, pairwise comparison bet. each 2 groups were done using **Post Hoc Test (Tukey)**

p: p value for comparing between the studied groups

*: Statistically significant at p ≤ 0.05

a: Significant with **Control**

b: Significant with **Quercetin control**

c: Significant with **AlCl3 (untreated).**

### Hepatic and renal expression of PGC-1α, mtTFA, Nrf2, and TNF-α

**Fig. 1:**
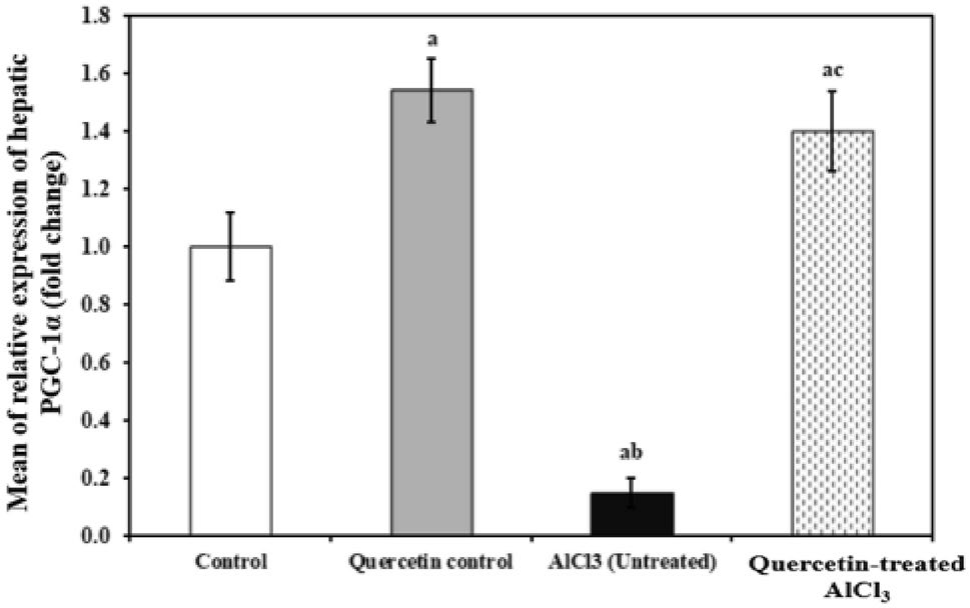
Hepatic PGC-1 α content (ng/mg protein) in the different studied groups.

**Fig. 2:**
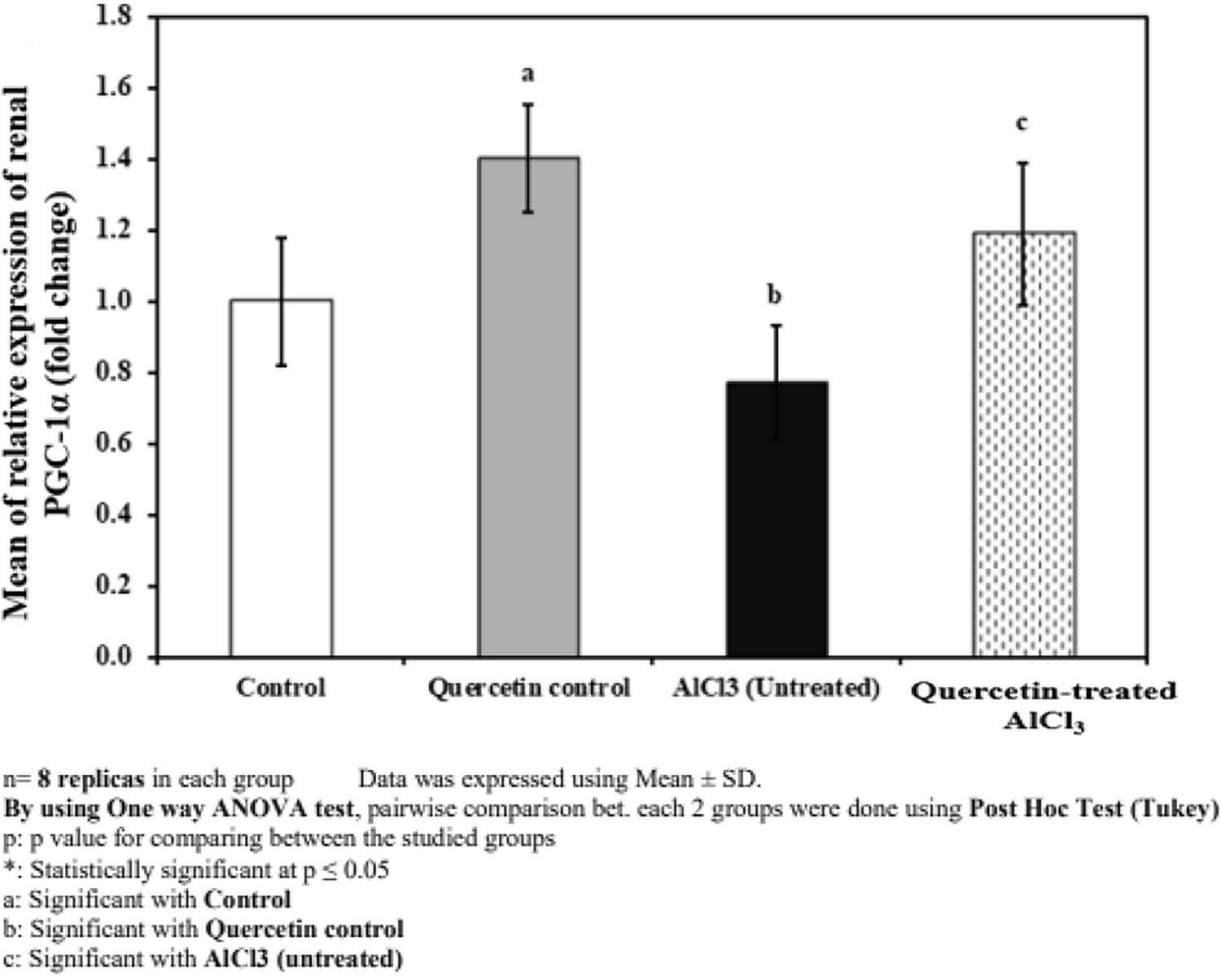
Renal PGC-1 α content (ng/mg protein) in the different studied groups.

**Fig. 3:**
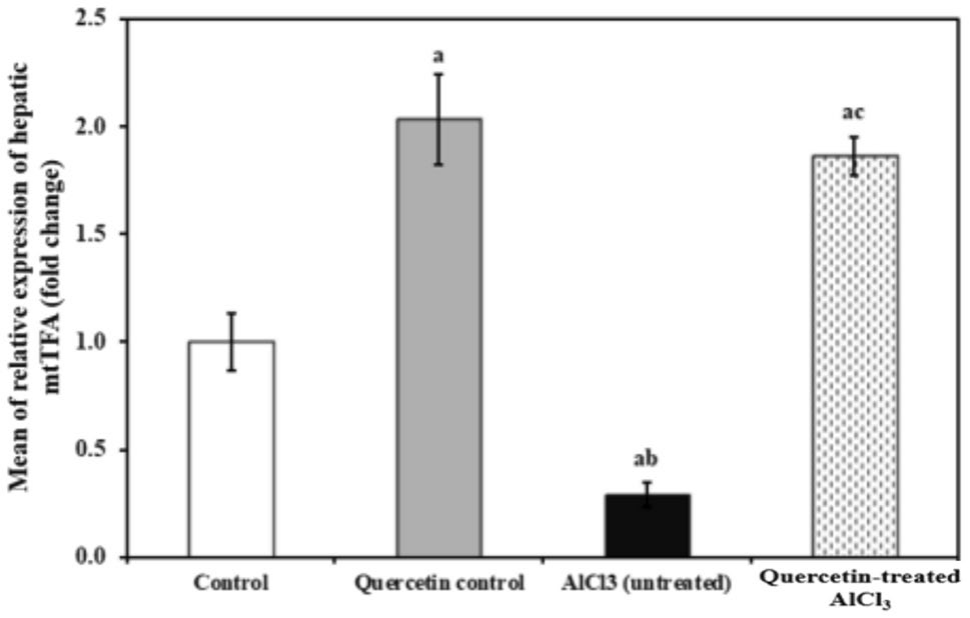
Hepatic mtTFA gene expression (Fold change) in the different studied groups.

**Fig. 4:**
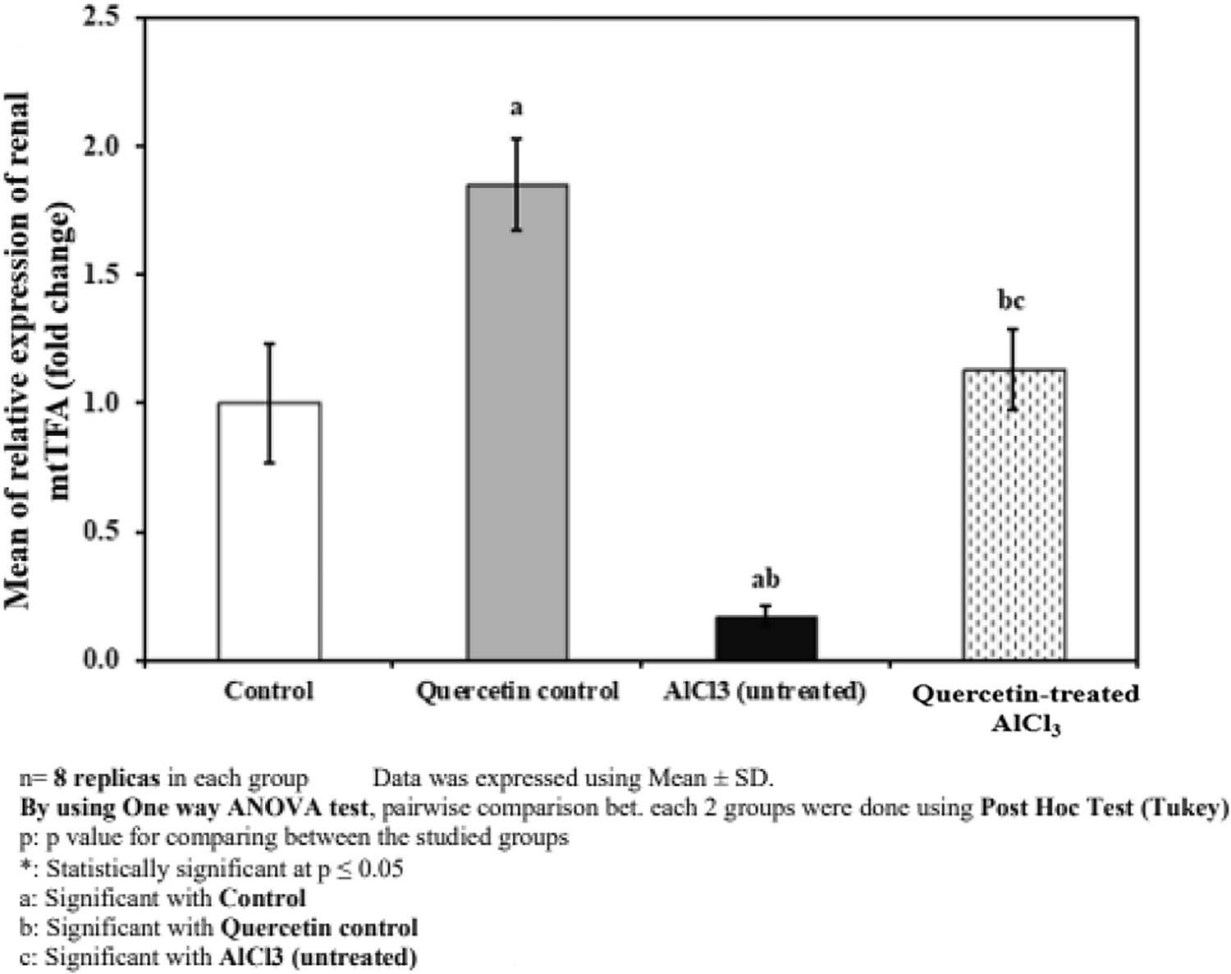
Renal mtTFA gene expression (Fold change) in the different studied groups.

**Fig. 5:**
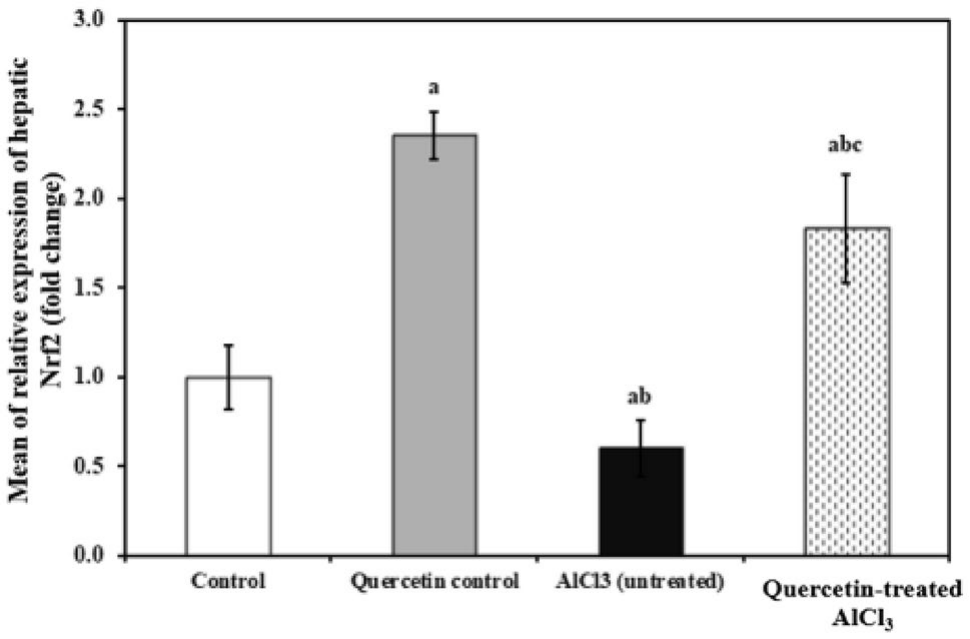
Hepatic Nrf2 gene expression (Fold change) in the different studied groups.

**Fig. 6:**
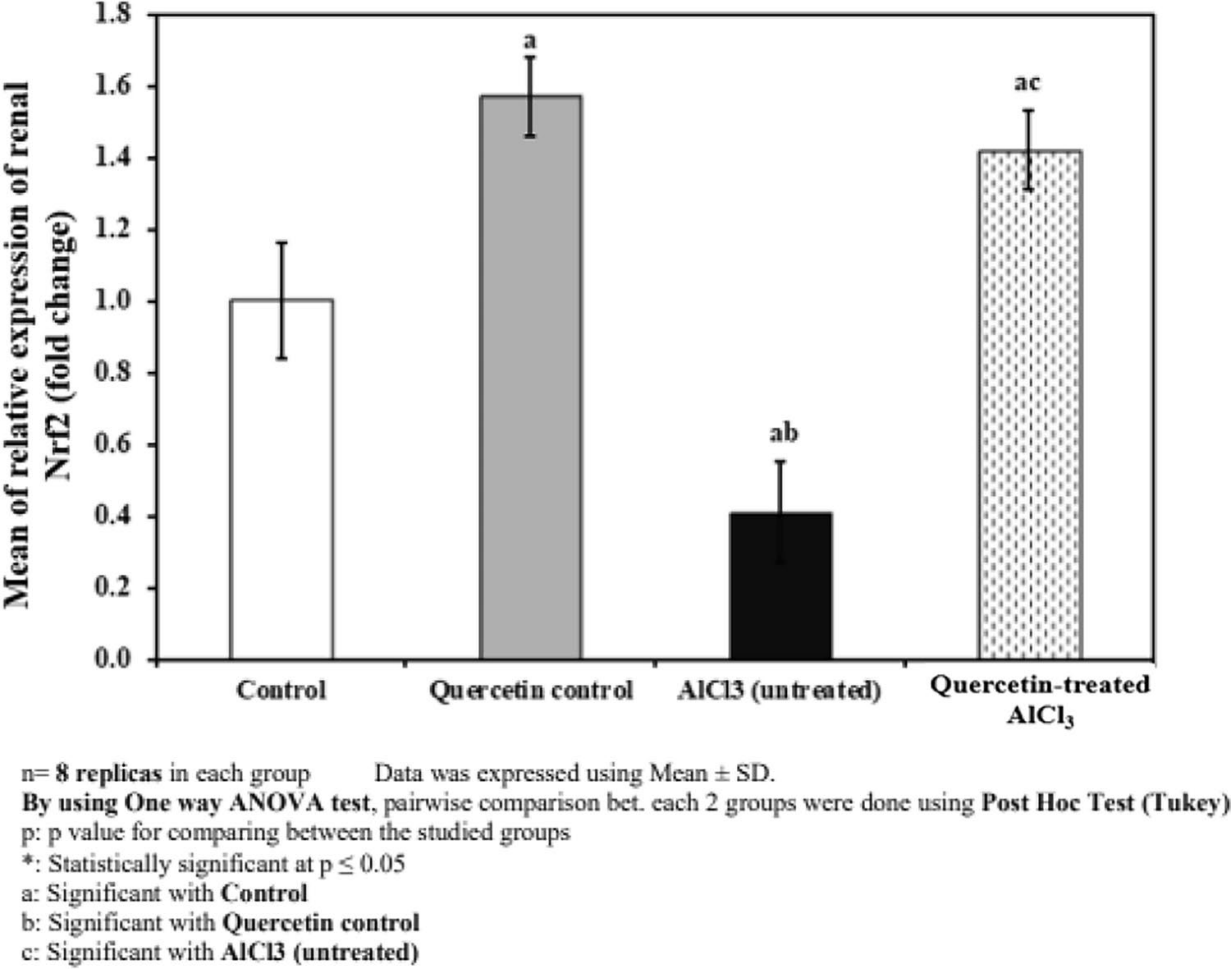
Renal Nrf2 gene expression (Fold change) in the different studied groups.

**Fig. 7:**
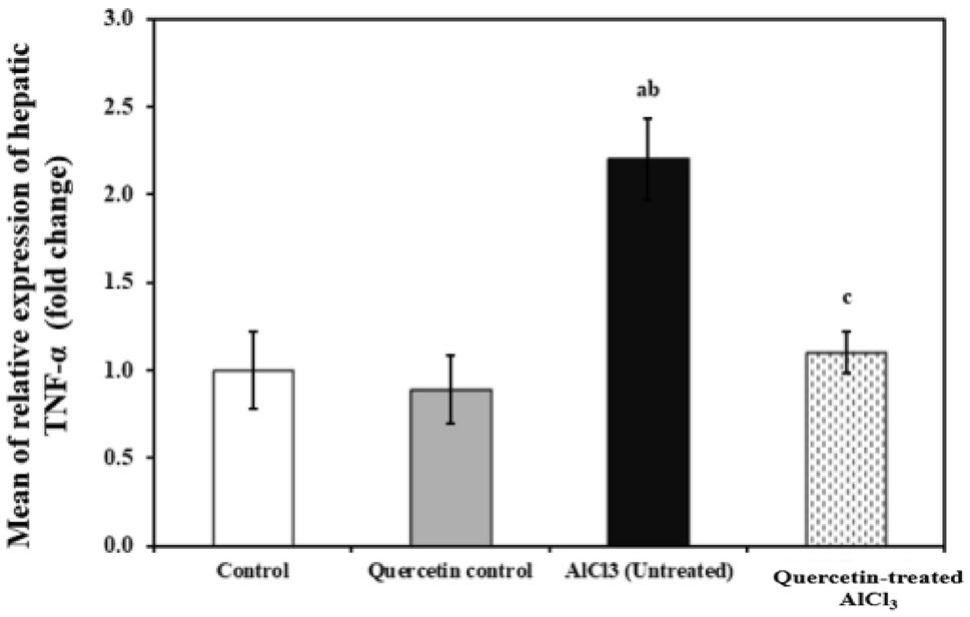
Hepatic TNF-α gene expression (Fold change) in the different studied groups.

**Fig. 8:**
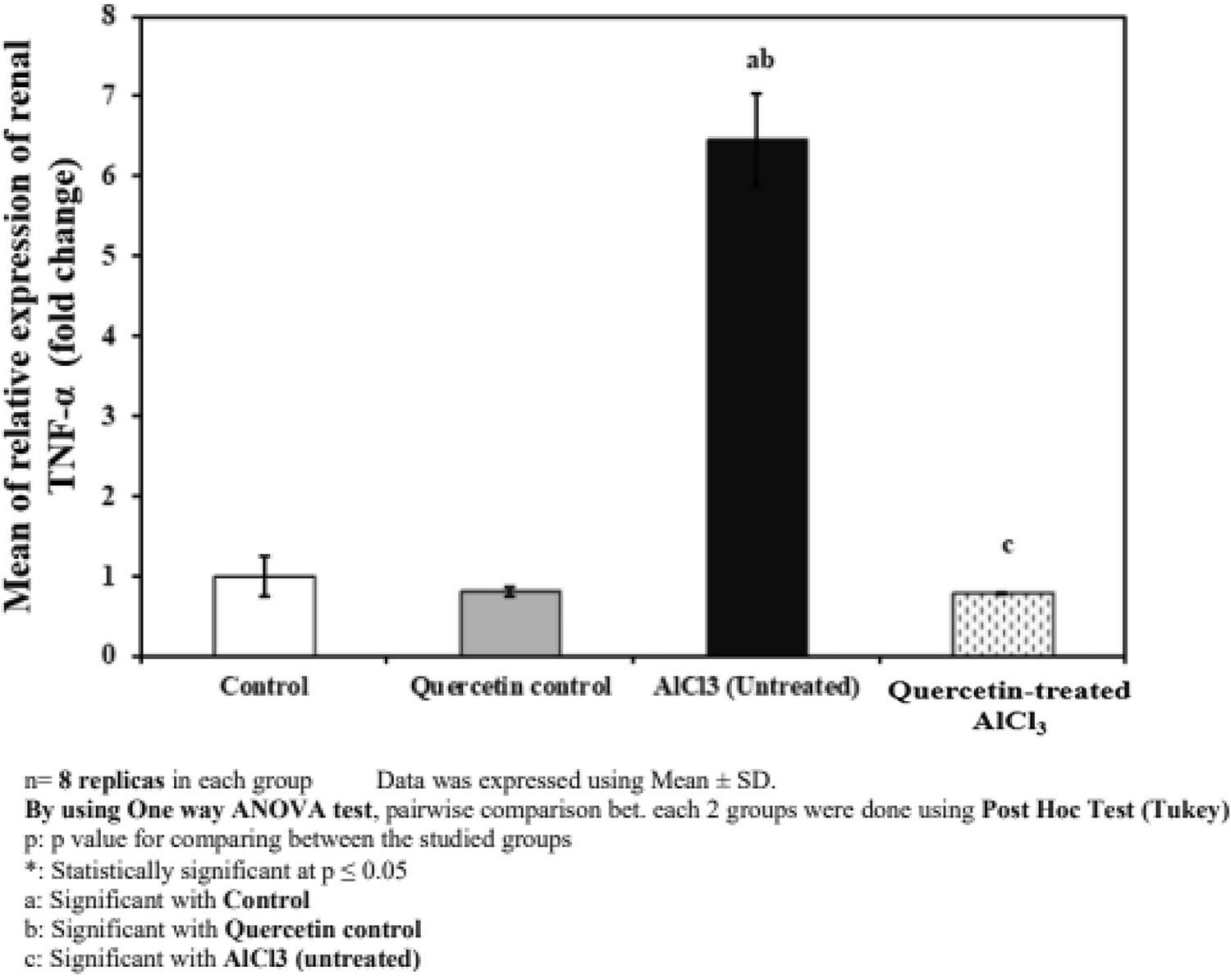
Renal TNF-α gene expression (Fold change) in the different studied groups.

As shown in (Figs. 1,2,3,4,5,6,7,8**),** AlCl_3_ exposure led to significant downregulation of genes related to mitochondrial function, such as PGC-1α, mtTFA, and Nrf2, while upregulating TNF-α expression (p ≤ 0.05). Quercetin treatment significantly restored the expression of these genes toward control levels (p ≤ 0.05), indicating its role in mitigating mitochondrial dysfunction and inflammation.

**Fig. 9:**
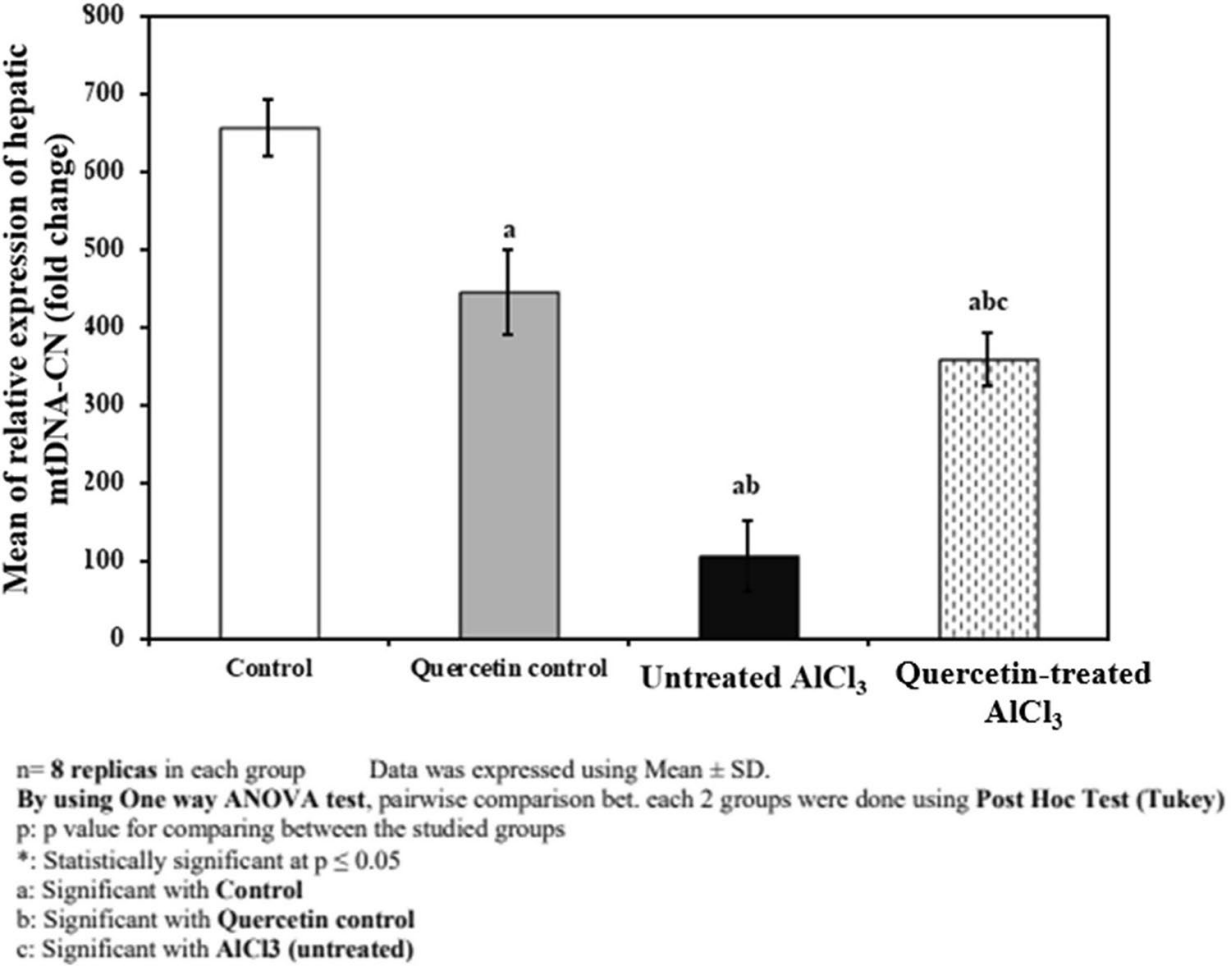
Hepatic mtDNA-CN in the different studied groups.

**Fig. 10:**
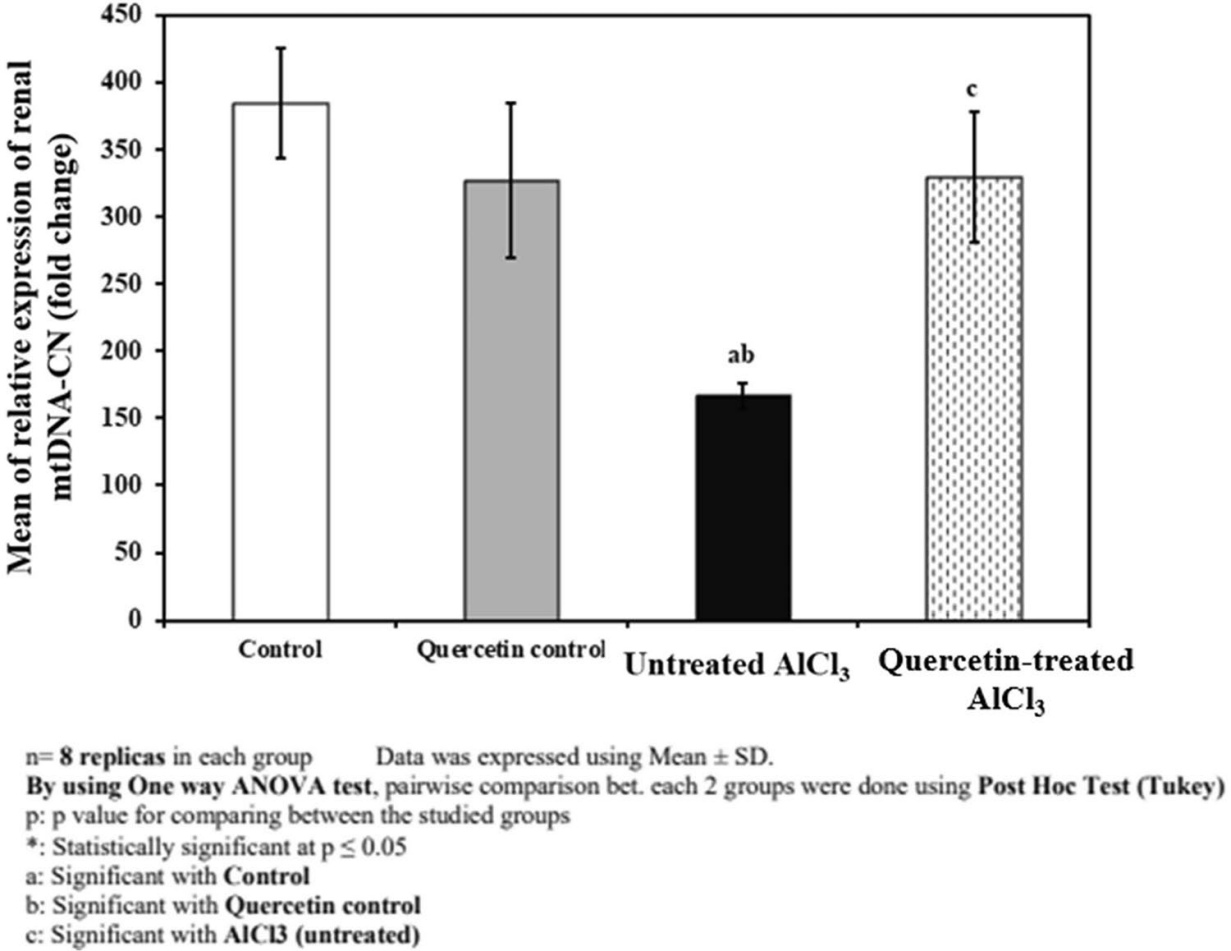
Renal mtDNA-CN in the different studied groups.

As shown in (Figs. 9,10**),** Mitochondrial DNA copy number (mtDNA-CN) was significantly reduced in AlCl_3_-exposed rats (p ≤ 0.05), but treatment with quercetin significantly increased mtDNA-CN levels (p ≤ 0.05).

### Correlation studies

#### Correlation between different parameters in hepatic tissues in quercetin-treated AlCl_3_ group

**Fig. 11:**
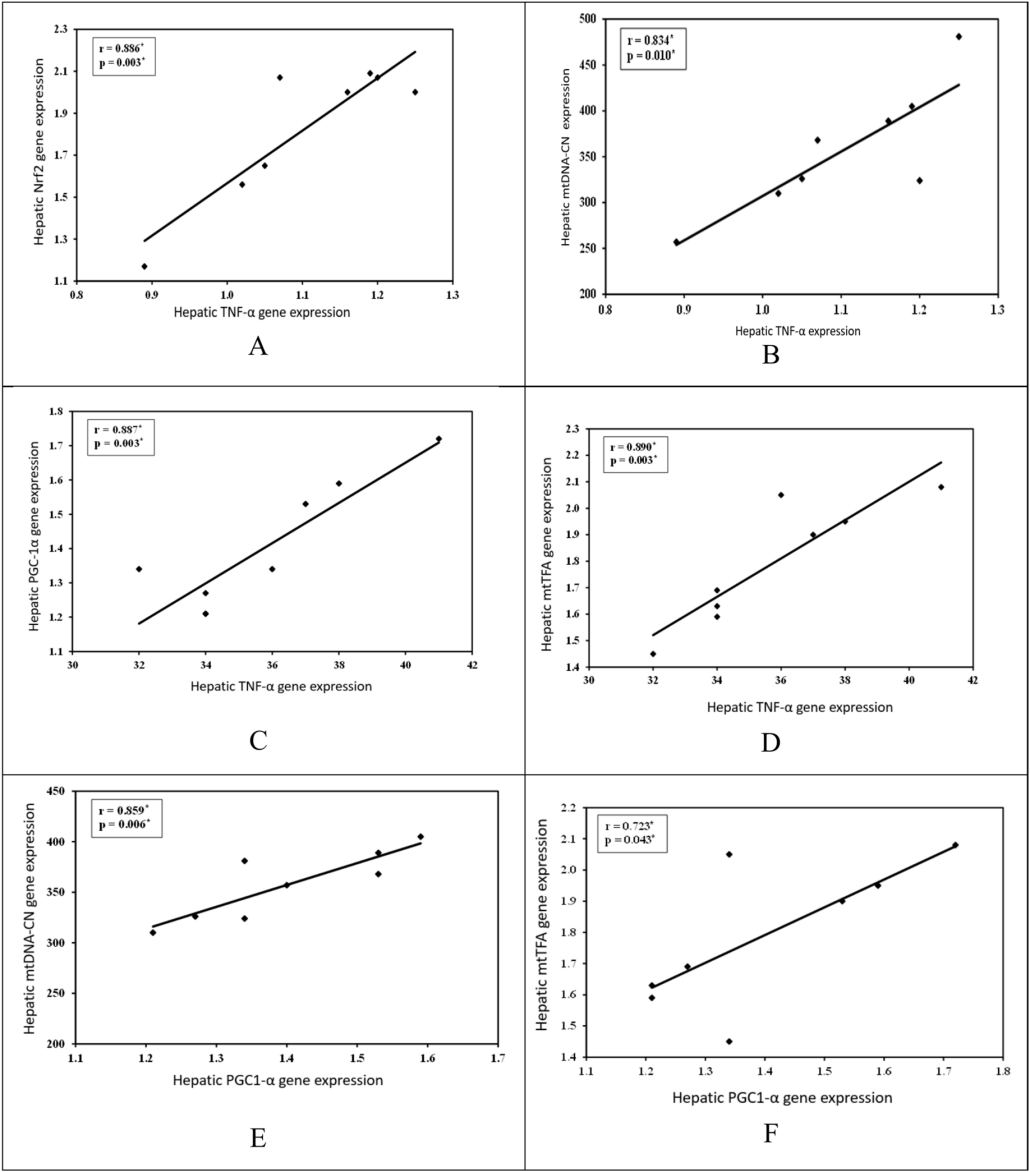
Hepatic correlation studies.

- Tumer necrosis factor-α (TNF-α) gene expression was positively correlated with Nrf2 gene expression (r= 0.886, p<0.003) **(**Fig. 11A**)**, mtDNA-CN levels (r= 0.834, p<0.010) **(**Fig. 11B**)**, PGC-1α gene expression (r= 0.887, p<0.003) **(**Fig. 11C**)** and mtTFA gene expression (r= 0.890, p<0.003) **(**Fig. 11D**)**
- Peroxisome proliferator activator receptor gamma-coactivator 1α (PGC-1α) gene expression was positively correlated with mtDNA-CN expression (r= 0.859, p<0.006) **(**Fig. 11E**)** and mtTFA gene expression (r= 0.723, p<0.043) **(**Fig. 11F**)**

#### Correlation between different parameters in renal tissues in quercetin-treated AlCl_3_ group

**Fig. 12:**
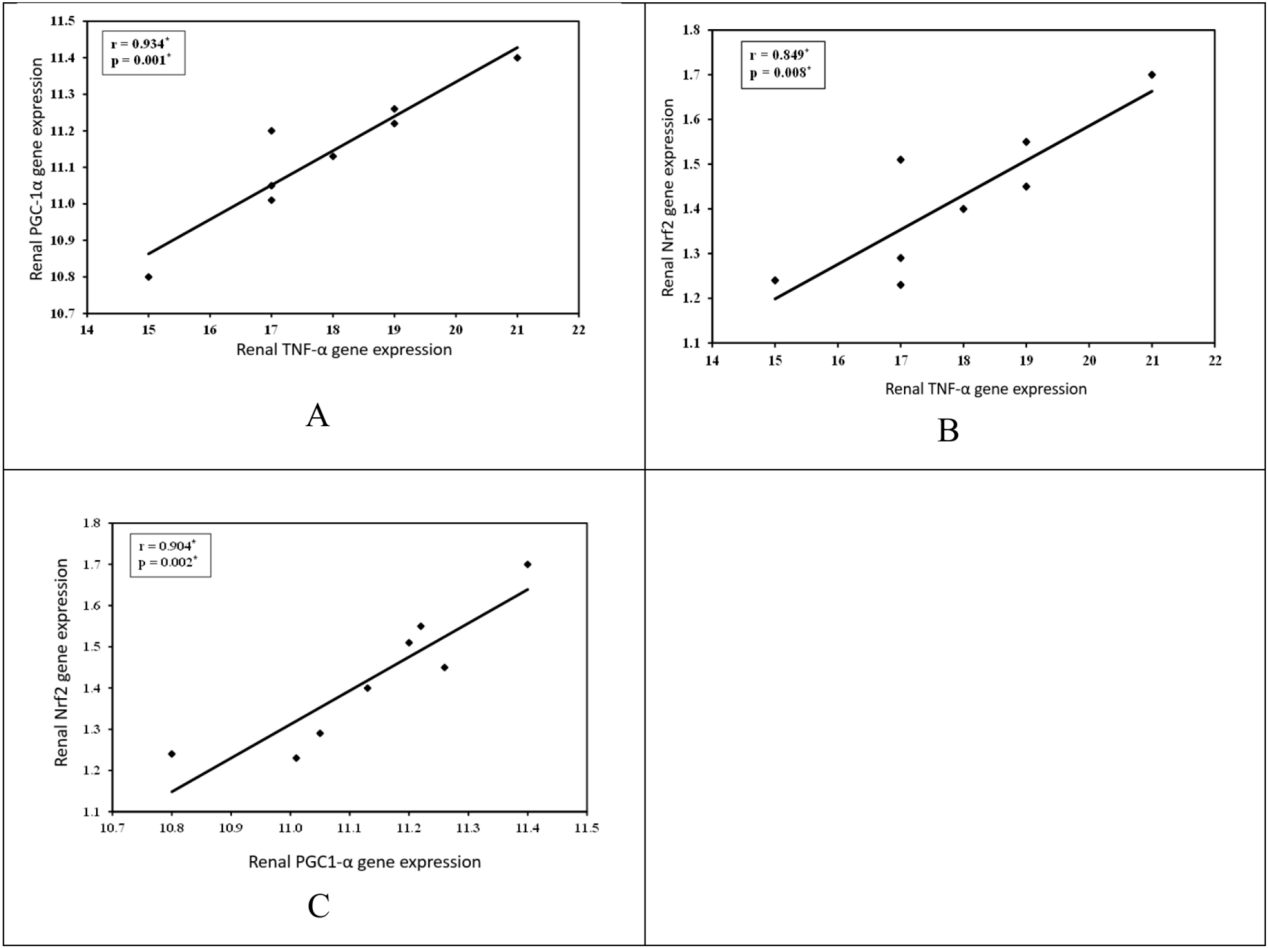
Renal correlation studies.

- Tumer necrosis factor-α (TNF-α) gene expression was positively correlated with PGC-1α gene expression (r= 0.934, p<0.001) **(**Fig. 12A**)** and Nrf2 gene expression (r= 0.849, p<0.008) **(**Fig. 12B**)**
- Peroxisome proliferator activator receptor gamma-coactivator 1α (PGC-1α) gene expression was positively correlated with renal Nrf2 gene expression (r= 0.904, p<0.002) **(**Fig. 12C**)**

### Histopathological analysis

#### Group I (Control group)

**Fig. 13:**
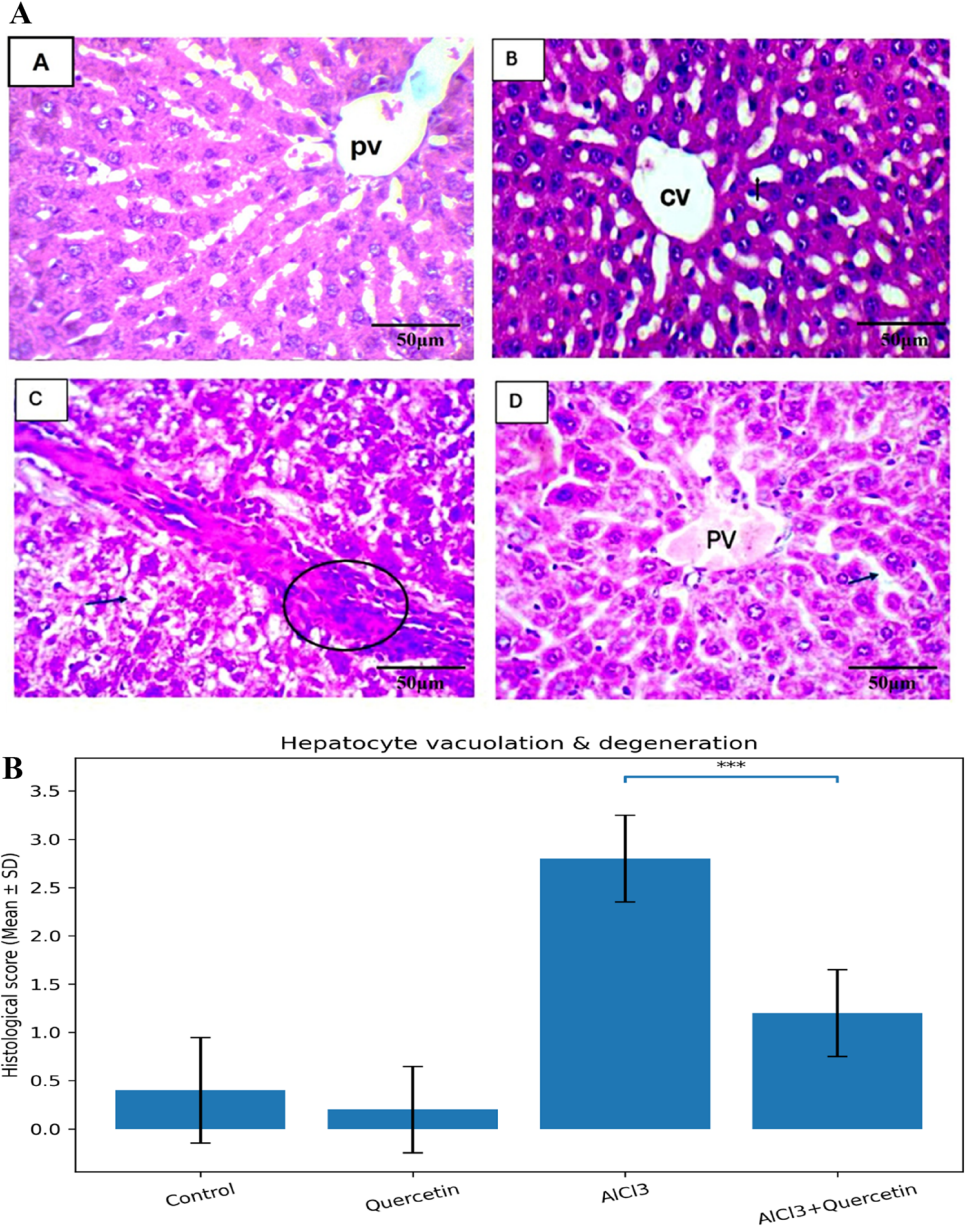
**A**: Microphotographs of liver tissue sections (H&E staining 400X, Bar 50μm). (**A**) control group showing normal histological structure of the liver with enlarged central vein (CV) and traditional hepatic lobules (**B**) Quercetin control group showing a well preserved hepatic architecture and dilatation of sinusoids observed ( ) **(C)** AlCl3 group (untreated) showing severe damage of hepatic architecture with observed cytoplasmic  vacuolation (  ) and formation of fibrous septa infiltrated with inflammatory cells (Circle) **(D)** AlCl3 group (treated) showing restoration of the normal histological structure of the liver with observed congestion of portal vein (PV) and dilatation of sinusoids ( ). **B**: Histological score of hepatocyte vacuolation and degeneration. AlCl3​ exposure significantly increased liver damage compared to the control, while Quercetin co-treatment (AlCl3​ + Quercetin) markedly reduced the histological score, showing its hepatoprotective effect.

**Fig. 14:**
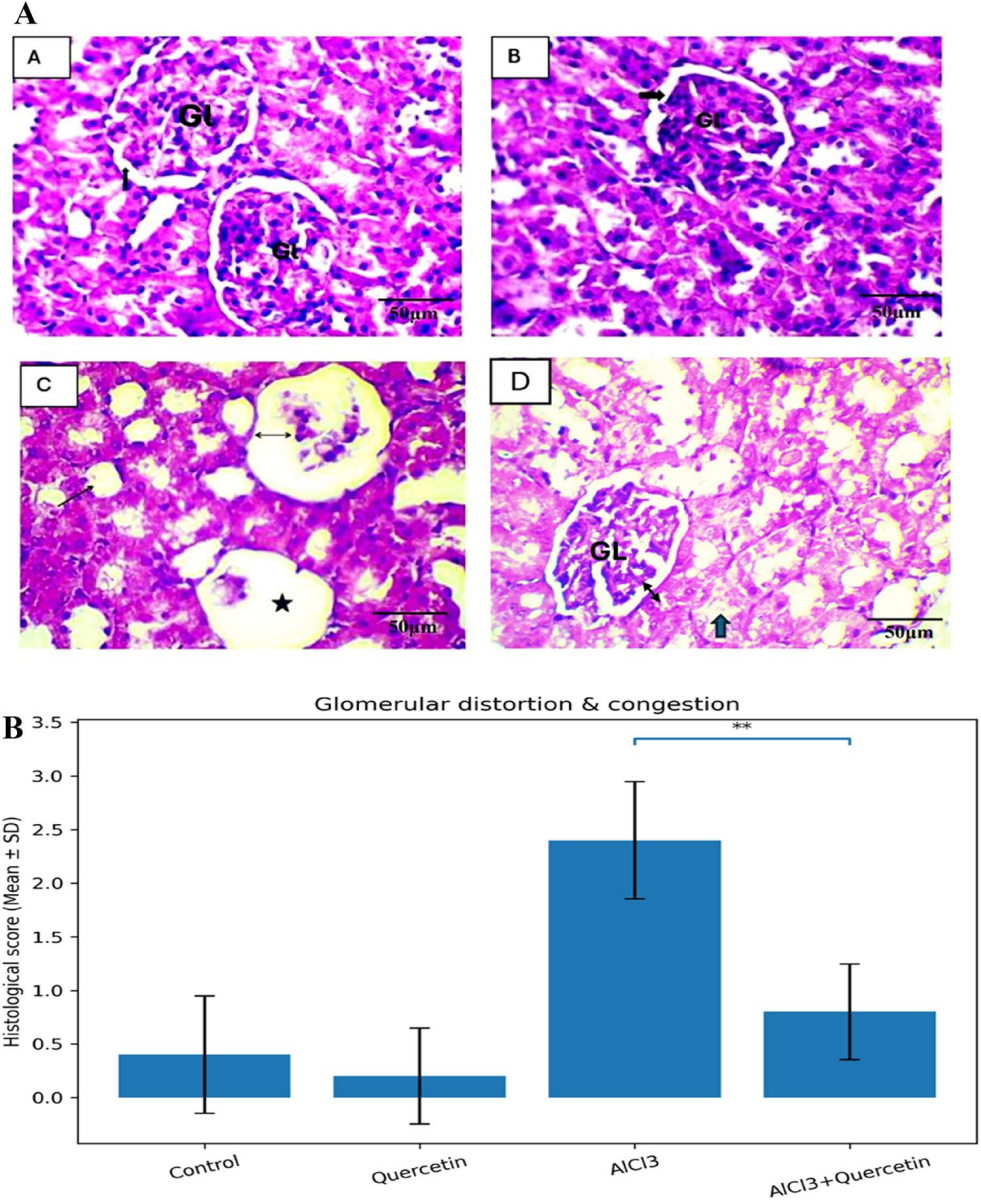
Microphotographs of kidney tissue sections (H&E staining 400X, Bar 50μm). (**A**) control group showing normal histological structure of the kidney with normal glomerulus size(GL) and intact bowman capsule space ( ) (**B**) Quercetin control group showing preserved histological structure of the kidney with normal GL size and intact bowman space ( ) (**C**) AlCl3 group (untreated) showing severe damage of kidney histological architecture with observed atrophied Glomerulus (star) with increased bowman capsule space () and dilatation of the tubules () (**D**) AlCl3 group (treated) showing restoration of the normal histological structure of the kidney with normal GL size and intact bowman space () with observed dilatation of kidney tubules (). **B**: Histological score of glomerular distortion and congestion in kidney. AlCl3​ exposure significantly increased kidney damage compared to the control, while Quercetin co-treatment (AlCl3​ + Quercetin) markedly reduced the histological score, showing its renal protective effect.

**In liver tissue:** Examination of sections obtained from liver of the control group showed normal histological structure. It was made up of traditional hepatic lobules with roughly hexagonal cells in shape with prominent nucleus and nucleolus observed, the nuclear cytoplasmic ratio was normal. Also, portal regions with connective tissue stroma appeared normal in structure. Moreover, normal visible central veins that are enlarged in some samples are observed (Fig. 13A)**. In kidney tissue:** Examination of sections obtained from kidney of the control group showed a thin connective tissue capsule with underlying renal cortex which contains a glomerulus and surrounding tubules with cuboidal epithelium. The majority of the cells in the proximal and distal convoluted tubules were of normal morphology (Fig. 14A).

#### Group II (Quercetin control group)

**In liver tissue:** Examination of sections obtained from liver of the quercetin control group did not show any sign of liver damage. The hepatic cells appeared normal with well-preserved cytoplasm. Hepatocytes were arranged in anatomizing cords, radiating outward from the center of each classic hepatic lobule (Fig. 13B).** In kidney tissue:** Examination of sections obtained from kidney of the quercetin control group showed normal histological structure. Of note, no prominent lesions in renal tubules are observed **(**Fig. 14B**).**

#### Group III (Untreated AlCl_3_)

**In liver tissue:** examination of sections obtained from liver of untreated AlCl_3_ group showed loss of hepatic architecture with prominent multinodular area of coagulative necrosis. Also, vacuolated cytoplasm with pyknotic nuclei and karyolitic cells observed. Moreover, significant haemorrhage with inflammatory cell infiltration in the portal and central vein area was also observed. What is perhaps more interesting is the appearance of fibrous septa with a shape of slender connective tissue band containing inflammatory cells indicating that AlCl_3_ induced fibrosis (Fig. 13C).** In kidney tissue:** microscopic examination of the kidney tissue of untreated AlCl3 group revealed severe damage and loss of normal kidney structure. There was a significant buildup of inflammatory cells in the interstitial spaces, and the glomeruli were significantly shrunken with obvious increase in Bowman’s capsule space **(**Fig. 14C**).**

#### Group IV (Quercetin-treated AlCl_3_ group)

**In liver tissue:** examination of sections obtained from liver of AlCl_3_ group treated with quercetin showed a marked reduction of morphological alteration and inflammation observed in untreated AlCl_3_ group. Moreover, a decline in cytoplasmic vacuolation is observed. Of note, no fibrous septa reported in the treated group indicating that quercetin attenuate the effect of AlCl_3_ in inducing necroinflammatory injury and fibrosis in the liver of treated rats (Fig. 13D). **In kidney tissue:** microscopic examination of the kidney tissue of AlCl_3_ group treated with quercetin showed remarkable histological improvement in the glomerular size observed along with a substantial reduction in interstitial infiltration of inflammatory cells. Of note, the tubular dilatation is reduced as well as the normal Bowman’s capsule space is restored **(**Fig. 14D**).**

## Discussion

Aluminum is a heavily utilized industrial and agricultural metal that is easily absorbed via the gastrointestinal system^29^**.** where it disrupts the intestinal barrier, increasing permeability and triggering apoptosis. Upon systemic absorption, AlCl_3_ acts as a potent hepatorenal toxin^30^^,^^9^**.**

In the present study, rats treated with AlCl_3_ solution exhibited a significant increase in serum ALT, ALP, AST activities, as well as urea, creatinine, and total bilirubin levels. Elevated serum ALP, ALT, and AST, alongside total bilirubin, serve as hallmark biomarkers of hepatotoxicity, with elevated bilirubin and ALP specifically indicating hepatobiliary injury and cholestasis^31^**.** The high serum activities of ALT and AST point to severe hepatocellular inflammation and injury; AlCl_3_ accumulation in hepatic tissue induces tissue necrosis, allowing these intracellular enzymes to escape into circulation^31^be^2^ Concurrently, elevated serum creatinine and urea reflect compromised renal function^32^**.** This aligns with previous reports that AlCl_3_ exposure damages the glomerulus, renal tubules, and renal cortex, thereby reducing glomerular filtration^33,34^**.**

Furthermore, AlCl_3_ administration disrupted lipid metabolism - a consequence of hepatic disease-induced metabolic alterations - leading to hyperglycemia, hypoproteinemia, hyperlipidemia, hypercholesterolemia, and hypertriglyceridemia^35,36^**.** The liver is central to lipid homeostasis, and its impairment here resulted in decreased HDL-C levels alongside increased triglycerides (TG), total cholesterol, and LDL-C^37^**.**

The present study suggests that Al exposure disrupts lipid metabolism, leading to increased serum triglycerides and total cholesterol levels in rats. This rise in cholesterol and ahypoactivity^37^ **.**

Quercetin treatment effectively reversed these pathologies, restoring liver function, reducing serum ALT, AST, ALP, and total bilirubin^38^**.** and decreasing serum urea and creatinine via oxidative stress reduction and the mitigation of renal injury pathways^39–41^. Quercetin also corrected the lipid profile by scavenging free radicals and normalizing lipid metabolism, significantly decreasing total cholesterol and TGs while increasing HDL-C^42^**.**

The underlying mechanism of AlCl_3_-induced hepatorenal toxicity relies heavily on the generation of reactive oxygen species (ROS) and subsequent oxidative stress, which severely compromises cellular and tissue health^2^**.** ^43^**.** In this study, AlCl_3_ exposure disrupted redox balance and induced profound lipid peroxidation, evidenced by a significant increase in malondialdehyde (MDA) content within both hepatic and renal tissues. This results line with previous studies^44^**.** This oxidative environment caused extensive macromolecular damage, indicated by significantly elevated serum advanced oxidation protein products (AOPPs)^45^**.** and increased hepatic and renal 8-hydroxy-2'-deoxyguanosine (8-OHdG) levels, confirming oxidant-mediated protein and DNA damage that impairs host antioxidant defenses^46^**.**

As a powerful natural dietary flavonoid, quercetin demonstrated robust hepatoprotective and nephroprotective effects against this acute organ damage^10,47–49^ **.** Quercetin’s unique chemical structure, featuring two benzene rings (specifically an active B ring), allows it to directly react with and scavenge excess ROS^50–53^**.** Moreover, it acts as a potent metal ion chelator, forming covalent bonds with Al ions to neutralize their charge, sequestering them within a stable complex, and preventing initial ROS generation^51,52,54,55^. Consequently, quercetin therapy markedly reduced MDA tissue content^56–59^**,** and^60^**.** diminished serum AOPPs^61^ and lowered 8-OHdG levels^62,63^ , thereby mitigating lipid peroxidation, safeguarding polyunsaturated fatty acids, and protecting cellular DNA

At the molecular level, AlCl_3_ exposure significantly downregulates the expression of the nuclear factor erythroid 2-related factor 2 (Nrf2) gene in both hepatic and renal tissues, that line with^45^**.** As a master transcription factor, Nrf2 preserves cellular homeostasis by encoding detoxification enzymes and activating vital antioxidant genes, including glutathione peroxidase (GPx), superoxide dismutase (SOD), and heme oxygenase-1^6,64,65^**.** The downregulation of Nrf2 by Al ions may stem from abnormalities in membrane receptors leading to intracellular ion accumulation^44^, which ultimately cripples the tissue’s antioxidant capacity. Depleted antioxidant defenses and elevated ROS further triggered inflammation, manifested by a significant increase in tissue tumor necrosis factor-alpha (TNF-α)^66^**.**

Conversely, quercetin treatment significantly upregulated Nrf2 expression and promoted its nuclear translocation in hepatic and renal tissues^67,68^ . While TNF-α-induced intracellular ROS caelhn naturally influence Nrf2 activation depending on ROS bioavailability under physiological conditions^69^. Quercetin acts as a targeted pharmacological activator of the Nrf2 signaling pathway^70^**.** By stimulating glutathione synthesis 70 and promoting the expression of antioxidant enzymes like catalase, GPx, and SOD, quercetin neutralizes ROS and re-establishes redox defenses.^71,72^**.**

Simultaneously, quercetin exhibited potent anti-inflammatory properties by markedly decreasing TNF-α expression in both liver and kidney tissues, protecting these vital organs from inflammatory damage^67,73^**.**

Mitochondrial dysfunction is another critical facet of AlCl_3_ toxicity. In this study, rats exposed to aluminum showed a marked downregulation of peroxisome proliferator-activated receptor-gamma coactivator 1-alpha (PGC-1α) and mitochondrial transcription factor A (mtTFA), culminating in a significant decrease in mitochondrial DNA copy number (mtDNA-CN)^74^**.** PGC-1α is the master regulator of mitochondrial biogenesis; it activates nuclear respiratory factors 1 and 2 (NRF-1/NRF-2) to initiate mtDNA transcription and replication, while simultaneously inhibiting oxidative damage^75^**.** By suppressing PGC-1α, AlCl_3_ aggravated the inflammatory response—causing a sharp increase in TNF-α expression—and severely destabilized the cellular redox balance^75^**.**

Our findings demonstrate that quercetin therapy in rats successfully counteracted this mitochondrial decay by upregulating PGC-1α mRNA and protein levels, mtTFA, and mtDNA-CN expression^3,76^**.** This can be explained in the light of previous studies which reported that, by reducing ROS, quercetin enhances PGC-1α expression, which activates NRF-1 and NRF-2. These factors prompt the expression of mtTFA, which translocate to the mitochondria, binds to mtDNA, and drives replication, thereby preserving mitochondrial morphology and density^3^**.** Furthermore, PGC-1α positively interacts with Nrf2 to regulate downstream antioxidant genes^77,78^**.**

Our biochemical and molecular findings are strongly corroborated by the histopathological alterations observed in both organs. In liver tissues, AlCl_3_-induced coagulative necrosis and inflammatory fibrous septa formation directly account for the leakage of ALT and AST into the serum, a structural damage driven by Nrf2 downregulation and TNF-alpha hyperactivation^2,45^. Quercetin treatment effectively restored hepatic architecture and eliminated fibrotic bands by upregulating the Nrf2 antioxidant pathway and suppressing inflammation^42^. Similarly, in renal tissues, the atrophied glomeruli, widened Bowman’s space, and tubular dilatation mirror the elevated serum urea and creatinine, resulting from mitochondrial decay and depressed PGC-1 α /mtTFA Pathways. Notably, Quercetin successfully reversed these renal cellular defects, confirming its role as a metabolic and structural stabilizer against aluminum-induced cytotoxicity^10^.

In the present study, there is a significant positive correlation of hepatic PGC-1α gene expression with hepatic mtTFA gene expression and hepatic mtDNA-CN. There is also a significant positive correlation of renal PGC-1α gene expression with renal Nrf2. Suggesting that quercetin triggers a highly coordinated, adaptive metabolic response that simultaneously upregulates mitochondrial biogenesis, suppresses inflammation, and neutralizes protein and oxidative damage under the stress of aluminum toxicity.

## Conclusion

This study demonstrates that quercetin effectively mitigates aluminum chloride-induced hepatorenal toxicity by reducing oxidative stress, inflammation, and mitochondrial dysfunction. The restoration of mitochondrial biogenesis markers and the improvement in histopathological outcomes highlight quercetin’s potential as a therapeutic agent in rats. These findings provide a strong foundation for future research aimed at developing quercetin-based interventions for aluminum-induced organ toxicity.

## Data Availability

Data will be available by request to the corresponding authors.

## Abbreviations

8-OHdG: 8-Hydroxy-2'-deoxyguanosine
AOPPs: Advanced oxidation protein products
ALP: Alkaline phosphatase
ALT: Alanine transaminase
AST: Aspartate transaminase
MDA: Malondialdehyde
mtDNA-CN: Mitochondrial DNA copy number
mtTFA: Mitochondrial transcription factor A
Nrf2: Nuclear factor-erythroid 2-related factor 2
PGC-1α: Peroxisome proliferator activator receptor gamma-coactivator 1α
TNF- α: Tumor necrosis factor-α
